# Supplementary figures and images for: Role of serine/threonine protein phosphatase PrpN in the life cycle of Bacillus anthracis
Source: PLoS Pathog. 2022 Aug 1;18(8):e1010729. doi: 10.1371/journal.ppat.1010729 (PMC9371265; doi:10.1371/journal.ppat.1010729)

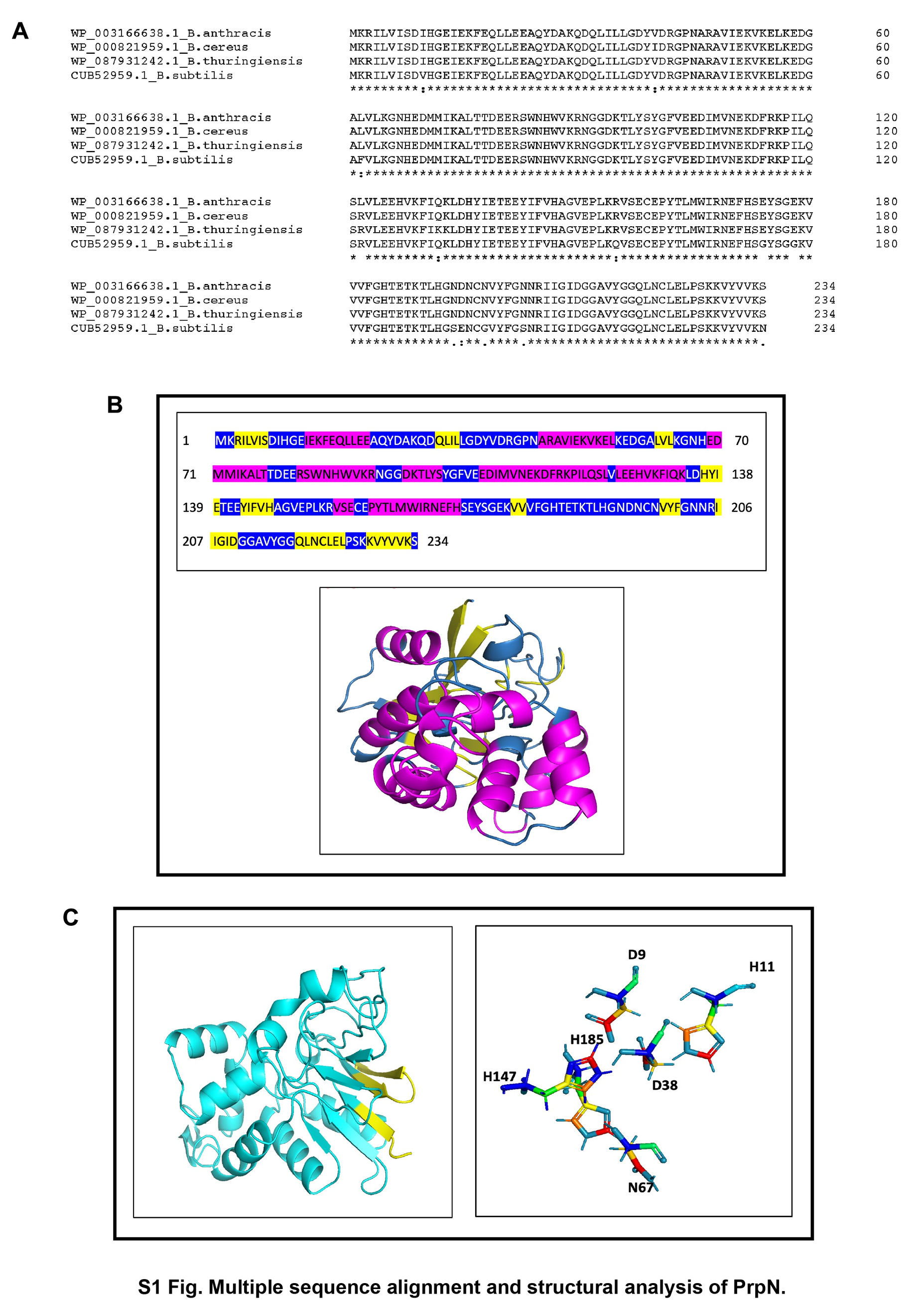

Supplement: S1 Fig — (A) Multiple sequence alignment of PrpN in the most studied Bacillus genus strains: B. cereus, B. thuringiensis and B. subtilis using Clustal Omega. “*” denotes perfect alignment, “:” denotes strong similarilty and “.” denotes weak similarity. (B) Structural overview of PrpN protein predicted using I-TASSER online server (Iterative threading assembly refinement). Primary amino acid sequence and structure of PrpN protein showing helix (pink colour), β-strands (yellow colour) and coils (blue colour). (C) PrpN structure depicting the phosphatase domain [metal-dependent protein phosphatase (MPP) family member] in cyan colour. Highly conserved metal-binding sites are indicated in the right panel. (TIF) [file ppat.1010729.s001.tif]

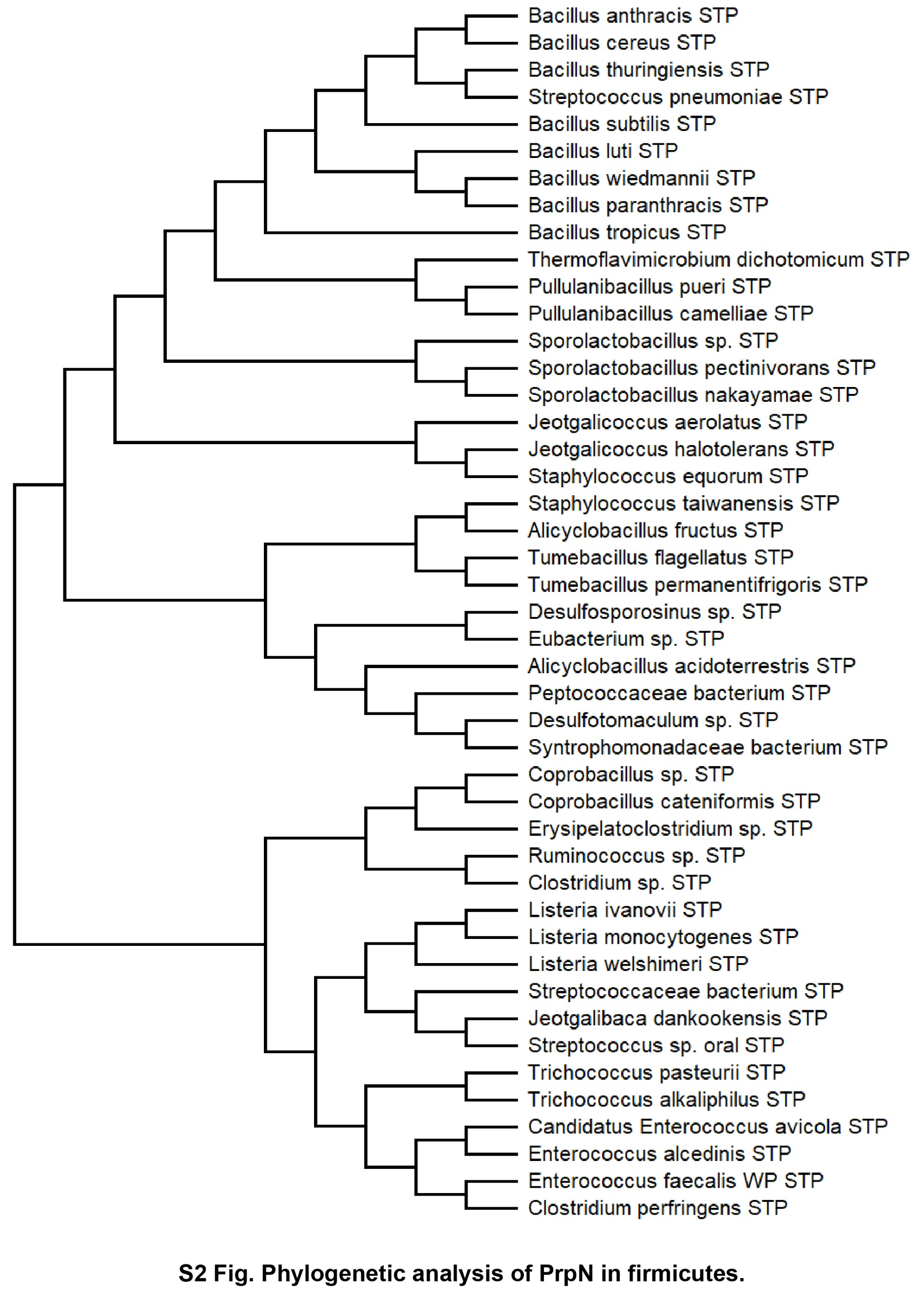

Supplement: S2 Fig — A phylogenetic tree representing the evolutionary relationship of PrpN in above-mentioned organisms. It was generated using Neighbor-Joining analysis conducted in MEGA XI. The tree is drawn to scale with branch lengths. This analysis involved 45 amino acid sequences. (TIF) [file ppat.1010729.s002.tif]

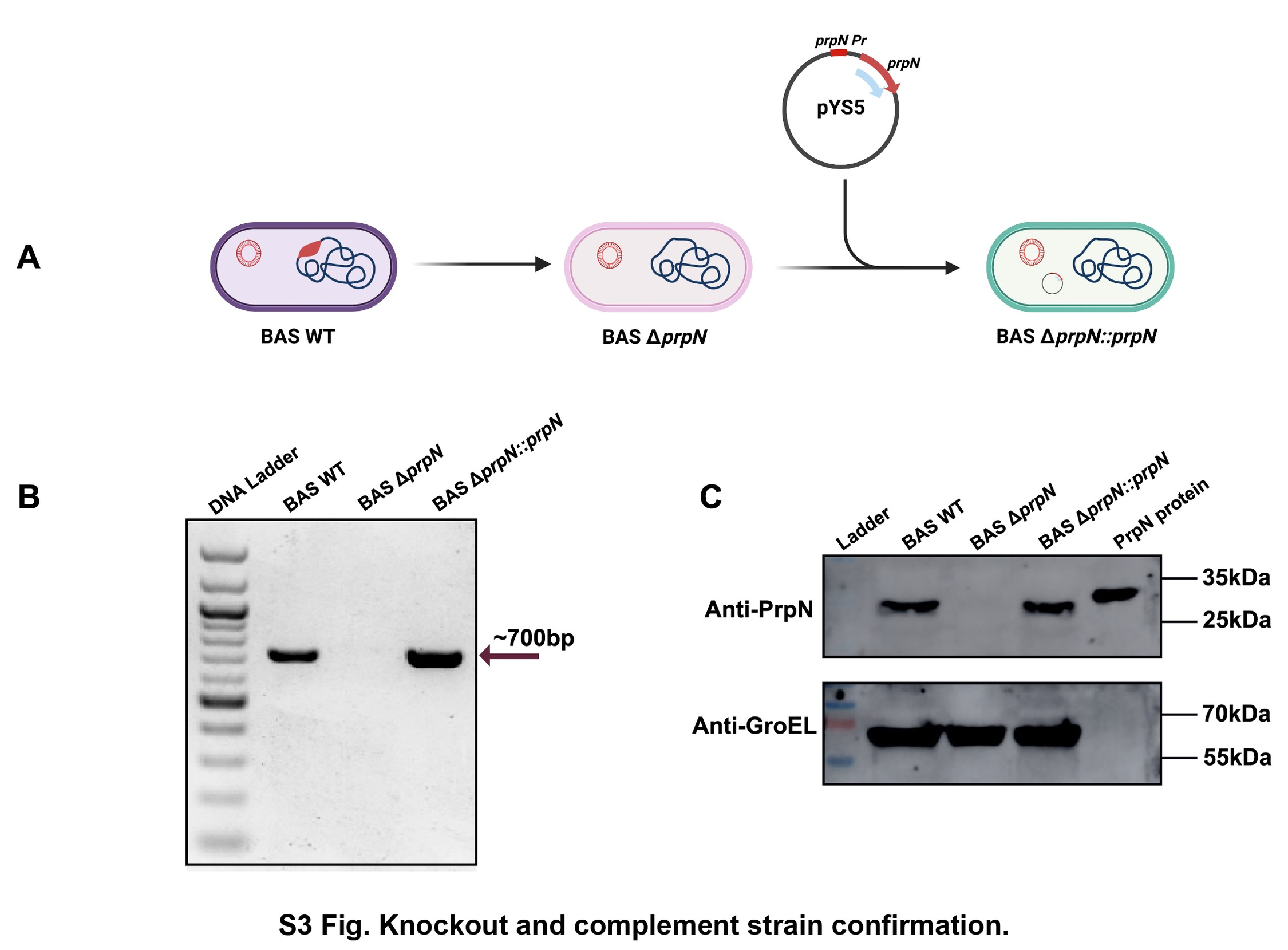

Supplement: S3 Fig — (A) Schematic representation of BAS ΔprpN and BAS ΔprpN::prpN strain generation. (B) Agarose gel showing PCR products amplified by prpN gene specific primers using indicated strains gDNA as template for strains’ confirmation at gene level. Ladder- 100 bp DNA Ladder H3 RTU (GeneDirex, Cat. No. SD003-R600). (C) Whole cell lysates of indicated strains were loaded in equal amount and probed using anti-PrpN and anti-GroEL for strains’ confirmation at protein level. Purified recombinant PrpN proteins with hexa-histidine tag was used as a positive control. Ladder- PageRuler Prestained Protein Ladder, Thermo-Scientific (Cat. No. 26616). (TIF) [file ppat.1010729.s003.tif]

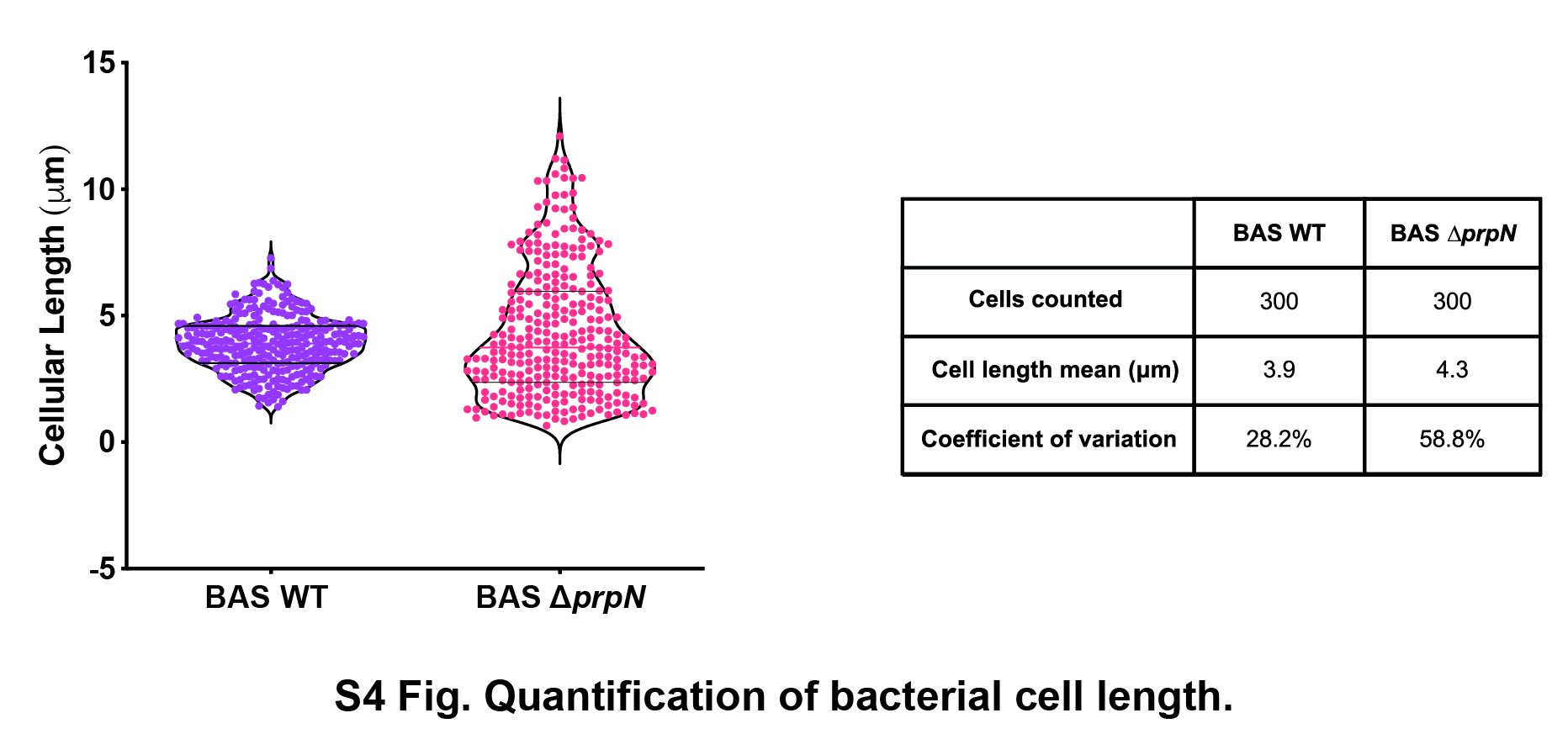

Supplement: S4 Fig — Bacterial cell length was measured using ImageJ software and the values were plotted as box and violin graph using GraphPad Prism software. All the data points (N = 300) are indicated in the graph and the corresponding mean cellular length and coefficient of variation is depicted in a table. (TIF) [file ppat.1010729.s004.tif]

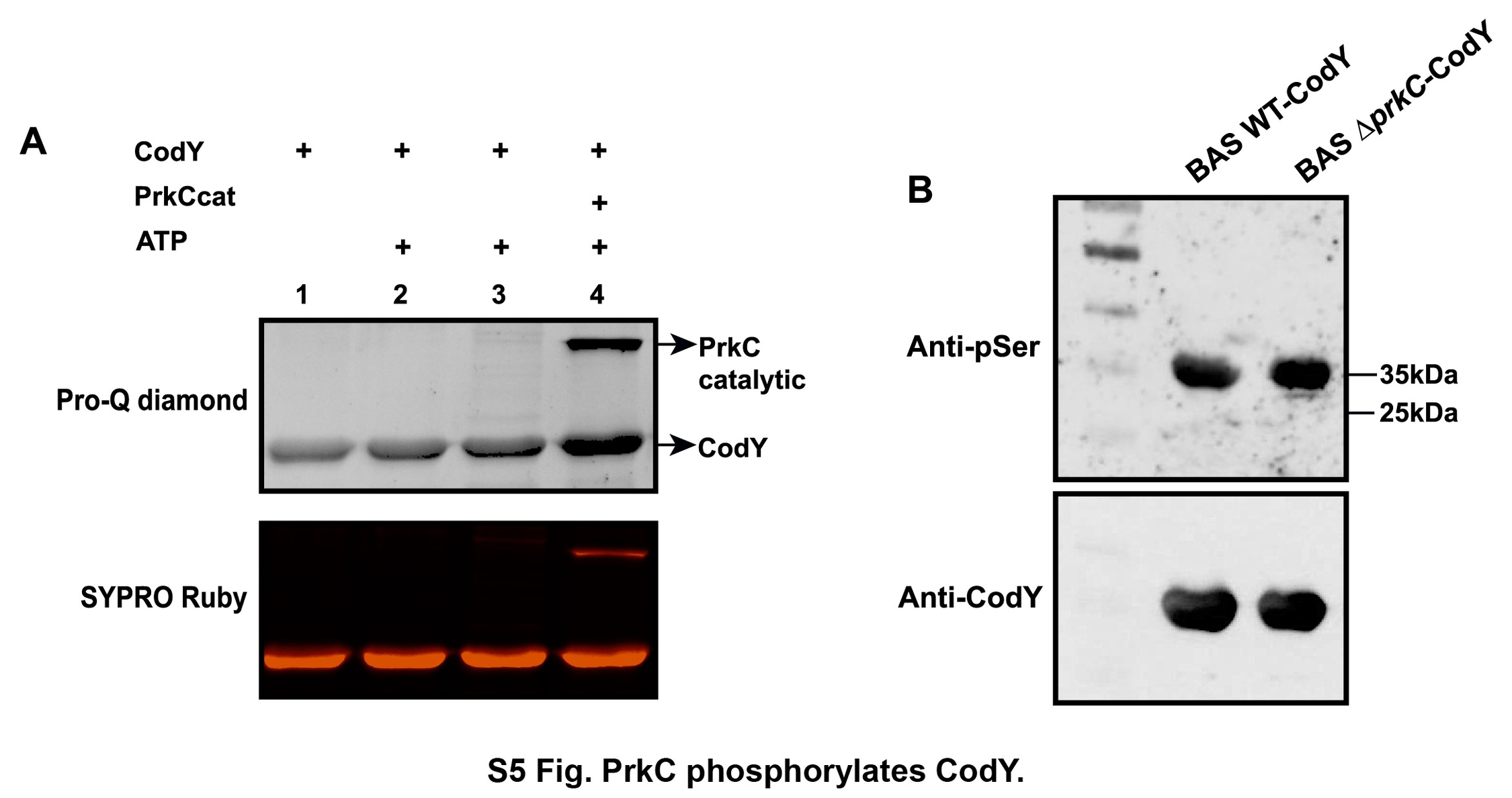

Supplement: S5 Fig — (A) In-vitro kinase assay was performed by incubating 2 μg CodY with 100 ng autophosphorylated PrkCcat. CodY alone and CodY incubated with ATP were taken as controls. The samples were resolved on 12% SDS PAGE and phosphorylation was visualized by using Pro-Q diamond phospho specific gel stain (upper panel) and SYPRO Ruby stain was used to visualize resolved proteins. (B) In-vivo PrkC mediated phosphorylation of CodY. CodY purified from indicated strains was loaded in equal amount and probed using anti-phosphoserine and anti-CodY. Indicated MWs were derived from adjacent lanes containing PageRuler Prestained Protein Ladder, Thermo Scientific (Cat. No. 26616). (TIF) [file ppat.1010729.s005.tif]

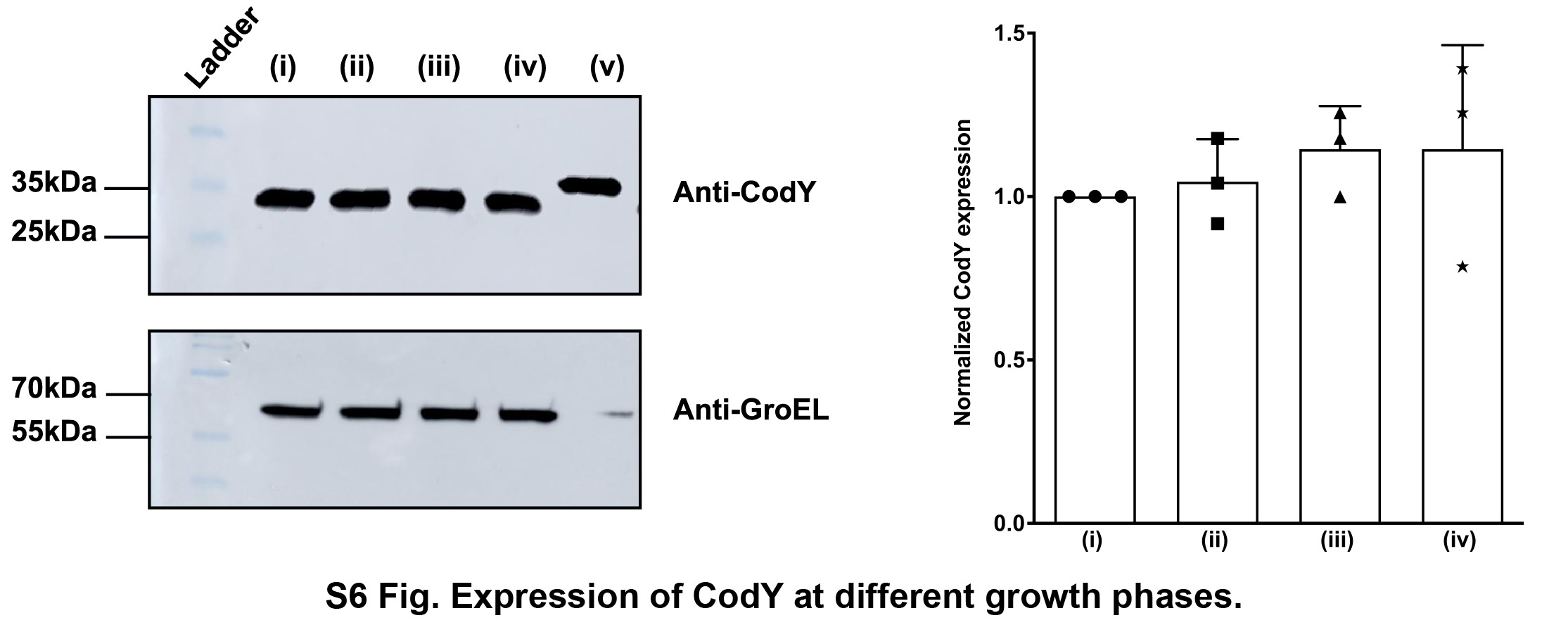

Supplement: S6 Fig — (Left panel) Representative immunoblot showing growth dependent differential expression of CodY in BAS WT strain. Equal amount of protein lysates prepared from different growth phases–(i) lag phase, (ii) exponential phase, (iii) early stationary phase and (iv) late stationary/sporulation initiation phase was loaded and probed using anti-PrpN and anti-GroEL. Lane (v) indicates purified recombinant CodY protein. Ladder- PageRuler Prestained Protein Ladder, Thermo-Scientific (Cat. No. 26616). (Right panel) Densitometer analysis were done using Amersham Imager600 software and the corresponding CodY/GroEL ratio were plotted in a bar graph using GraphPad Prism. Densitometer readings calculated from three experiments executed independently are shown in the bar graph. (TIF) [file ppat.1010729.s006.tif]

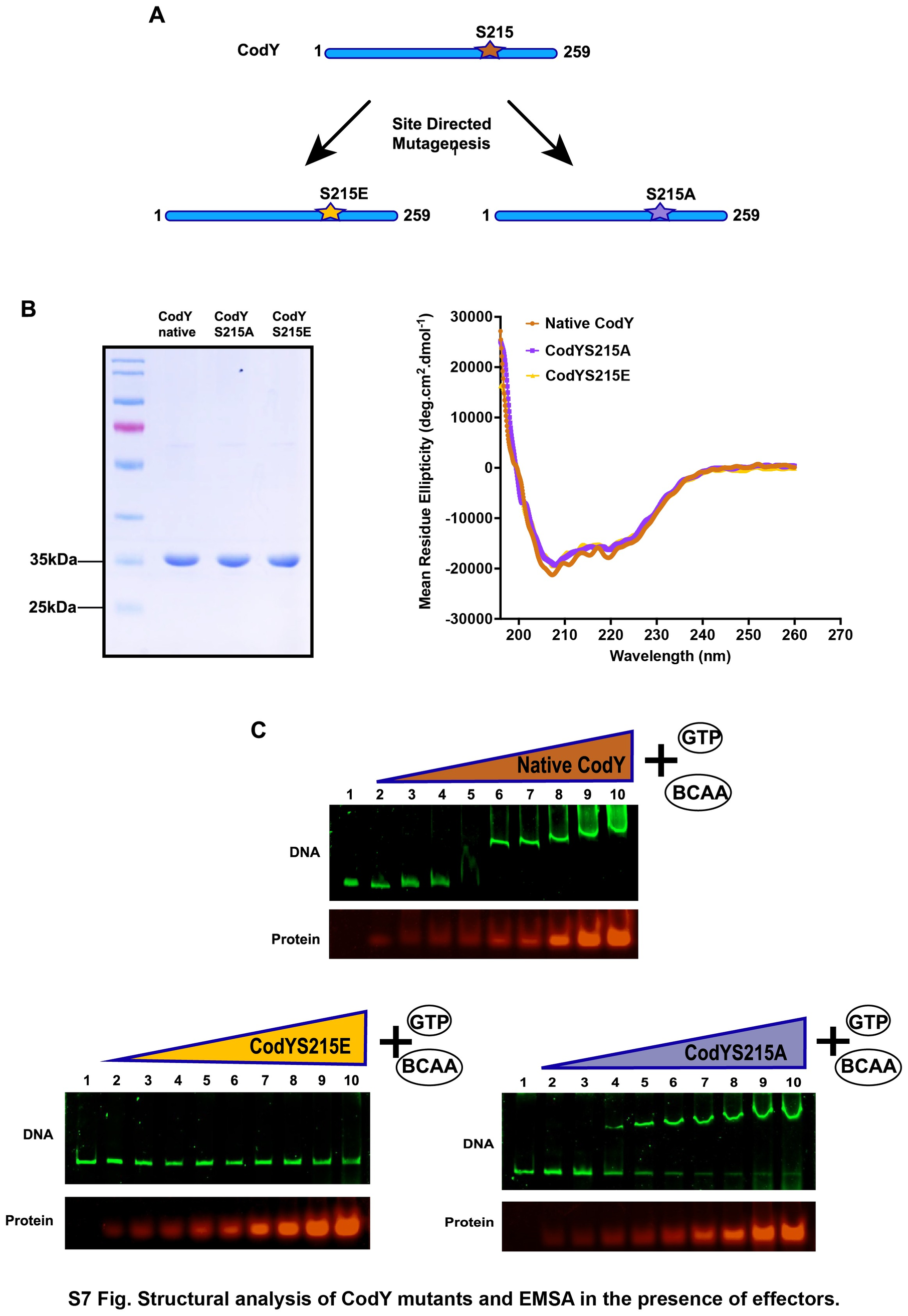

Supplement: S7 Fig — (A) Schematic illustration of the strategy followed for CodY mutant generation. (B) Coomassie stained SDS-PAGE of recombinant native CodY, CodYS215A and CodYS215E proteins (left panel). Superimposed CD spectrum of native and mutant CodY proteins (right panel). (C) Electrophoretic Mobility-Shift Assay using SYBR Green and SYPRO Ruby stains in the presence of effector molecules. Increasing amounts (1, 1.5, 3, 4, 5, 6, 8, 10 and 12μM) of native CodY, CodY S215E and CodY S215A proteins were added in a binding reaction containing 4 nM atxA promoter region as probe in the presence of GTP and BCAA. Lane 1 represents only DNA control. Upper gel image represents DNA bands stained using SYBR Green and lower image represents protein bands stained using SYPRO Ruby. (TIF) [file ppat.1010729.s007.tif]

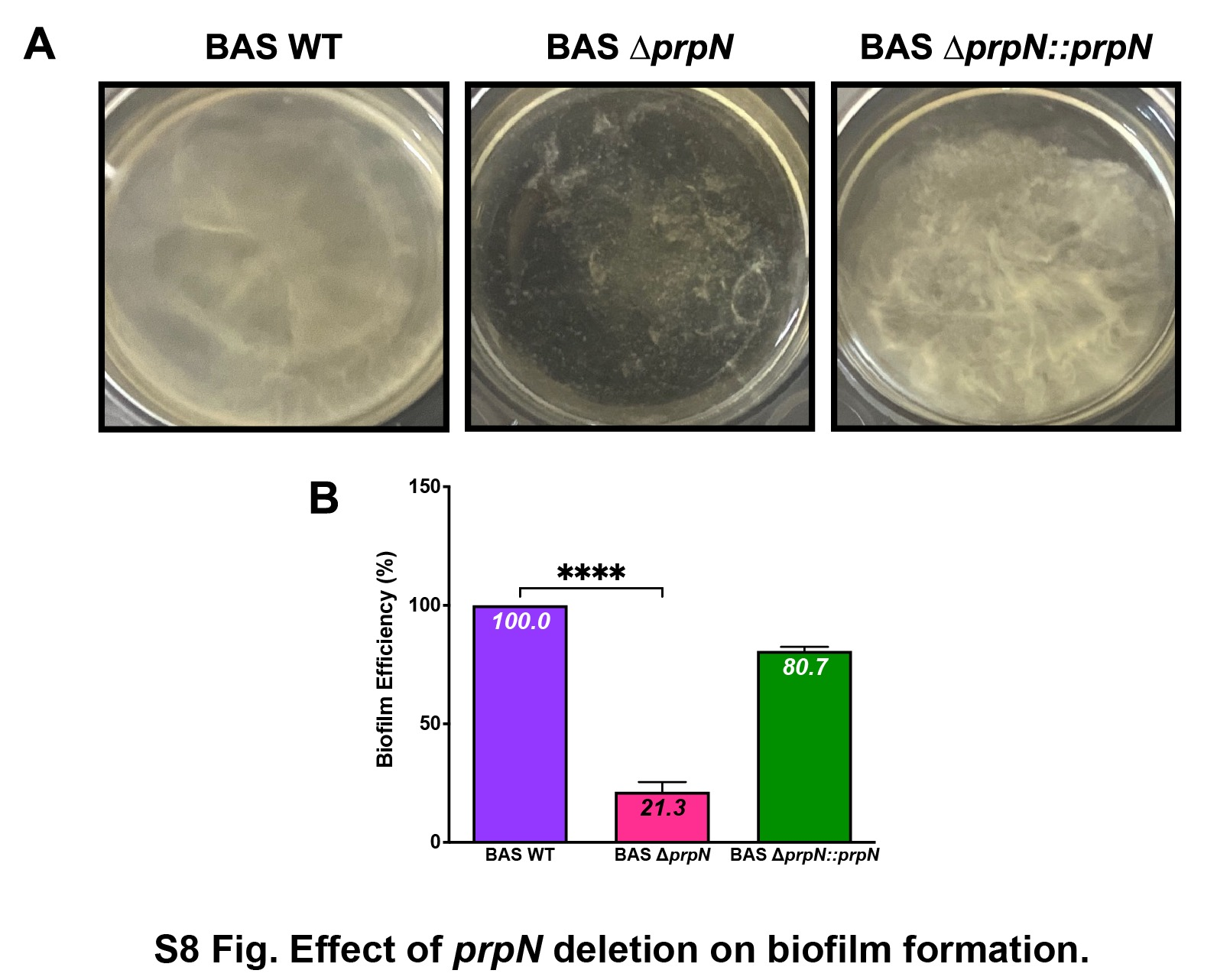

Supplement: S8 Fig — (A) Representative images of BAS WT, BAS ΔprpN and BAS ΔprpN::prpN biofilm formation in a 6-well plate. (B) Biofilm formation efficiency of these strains was estimated using crystal violet stain and were plotted w.r.t. BAS WT. Mean and standard error mean from six independent experiments are shown in the bar graphs. Statistical Analysis: Asterisks indicate statistical significance of the data set calculated using two-tailed Student’s t test. * corresponds to p<0.05; ** corresponds to p<0.01; *** corresponds to p<0.001 and **** corresponds to p<0.0001. (TIF) [file ppat.1010729.s008.tif]

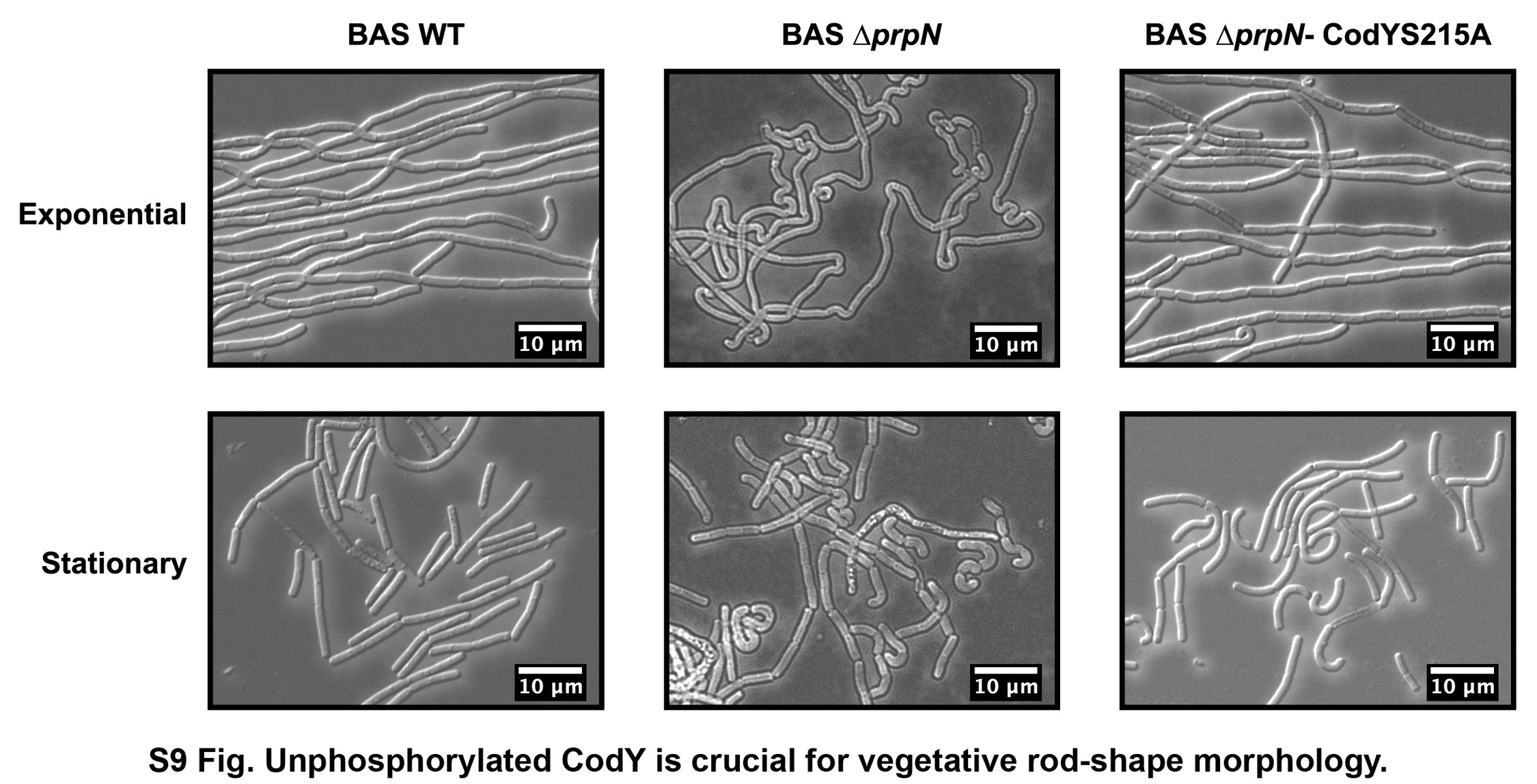

Supplement: S9 Fig — Representative phase contrast microscopy images of BAS WT, BAS ΔprpN and BAS ΔprpN::codYS215A strains at different time points. Scale bars are depicted in the images. (TIF) [file ppat.1010729.s009.tif]
