# Supplementary material for: Role of serine/threonine protein phosphatase PrpN in the life cycle of Bacillus anthracis
Source: PLoS Pathog. 2022 Aug 1;18(8):e1010729. doi: 10.1371/journal.ppat.1010729 (PMC9371265; doi:10.1371/journal.ppat.1010729)
Supplement: S1 Table — (PDF) [file ppat.1010729.s010.pdf]

S1 Table. List of primers used in this study	
**Primer Name**	**Primer sequence 5´→ 3´**
**Primers for PrpN operon prediction**	
OC FP1	GCGACGAAACAATCTCCAGC
OC RP1	GCTGGCCACCATATACAGCA
OC FP2	GCCCTCGTTCTAAAGGGCAA
OC RP2	CGAATCCCCAGACACGATCAA
**Primers for PrpN knockout and complemented strain**	
Single crossover LFP	GGGG*CTCGAG*GCAAAGTGGCATACTGCAACAGG
Single crossover LRP	GGGG*ACTAGT*CCTTACAACAATGTAAGTCTCTTGTCATCC
Single crossover RFP	GGGG*CTCGAG*GGTGGTGCTGTATATGGTGGCCAGC
Single crossover RRP	GGGG*ACTAGT*GCTTCATACTTTGTCCATCTACTTGAAC
PrpN Internal FP	GAAGATGGAGCCCTCGTTC
PrpN Internal RP	CATTACAATTATCATTTCCATGAAGCG
PrpN Flanking FP	AAAAAAGGATACGGCTTTCTT
PrpN Flanking RP	TCTAAAGAATCTCTCGCTGTCAA
PrpN native pro FP (*KpnI*)	CCCC*GGTACC*AAAAAAGGATACGGCTTTCTTTAGGAG
PrpN native pro RP (*SmaI*)	GGGG*CCCGGG*TCAACTTTTTACTACGTACACCTTTTTAC
**CodY SDM Primers**	
CodY FP (*SpeI*)	CCC*ACTAGT*CATGGAATTATTAGCAAAAAC
CodY RP (*BamHI*)	CCC*GGATCC*TTAGTTTGTTTTTAATTTAGC
CodY *S215E* FP	GCGTAGGAATCACTCGT*GAG*GTAATCGTAAATGCACTTCG
CodY *S215E* RP	CGAAGTGCATTTACGATTAC*CTC*ACGAGTGATTCCTACGC
CodY *S215A* FP	GCGTAGGAATCACTCGT*GCG*GTAATCGTAAATGCACTTCG
CodY *S215A* RP	CGAAGTGCATTTACGATTAC*CGC*ACGAGTGATTCCTACGC
**EMSA Primers**	
AtxA Promoter FP	GCTATTTCAATAGAAGAAACAAAAAACCAA
AtxA Promoter RP	CGATGGATATCGGTGTTAGCATGTC
**Real time PCR primers**	
AtxA RT FP	TGCATTTTGAAACTCAACGTATGC

AtxA RT Rp	ACGTTTTTCAAGCAGTGCAGA
RpoB RT FP	AACTTGCGCACATGGTTGAC
RpoB RT RP	CTGTCCACCGAACTGAGCTT
PrpN RT FP	CCAAATGCGCGTGCAGTAAT
PrpN RT RP	TGAACGTTCCTCATCAGTCGT
**Primers for Protein Purification**	
PrpN FP (*SacI*)	CACC*GAGCTC*ATGAAAAGAATTCTTGTTAT
PrpN RP (*NotI*)	CACC*GCGGCCGC*TCAACTTTTTACTACGTACA
PrpC FP (*BamHI*)	CACC*GGATCC*CCATGAAAGCCGTGTTTCTATC
PrpC RP (*NotI*)	CACC*GCGGCCGC*TCACCTACTTTCGTTTGTCGA
AtxA FP (*BamHI*)	CACC*GGATCC*CCATGCTAACACCGATATCCA
AtxA RP (*XhoI*)	GGG*CTCGAG*TTATATTATCTTTTTGATTTCATG
CodY FP (*BamHI*)	CCC*GGATCC*CCATGGAATTATTAGCAAAAAC
CodY RP (*XhoI*)	GGG*CTCGAG*TTAGTTTGTTTTTAATTTAGC
CodY RP with His$_6$-tag (*BamHI*)	CCC*GGATCC*TTAGTGGTGGTGGTGGTGGTTTGTTTTTAATTTAGC
PrkCcat FP (*BamHI*)	CACC*GGATCC*CCGTGCTGATTGGAAAACGCTTAAATG
PrkCcat RP (*XhoI*)	GGG*CTCGAG*TTACGCAGTTTCAATATCTCGTTTCATCGC
**Primers for Dual Expression (pETDuet)**	
CodY pETDuet FP (*BamHI*)	CCC*GGATCC*CATGGAATTATTAGCAAAAACAAG
CodY pETDuet RP (*SacI*)	CCC*GAGCTC*TTAGTTTGTTTTTAATTTAGC
PrkCcat pETDuet FP (*NdeI*)	GGGG*CATATG*GTGCTGATTGGAAAACGCTTAAATG
PrkCcat pETDuet RP (*KpnI*)	CCCC*GGTACC*CGCAGTTTCAATATCTCGTTTCATCGC
