# Supplementary material for: Role of serine/threonine protein phosphatase PrpN in the life cycle of Bacillus anthracis
Source: PLoS Pathog. 2022 Aug 1;18(8):e1010729. doi: 10.1371/journal.ppat.1010729 (PMC9371265; doi:10.1371/journal.ppat.1010729)
Supplement: S2 Table — (PDF) [file ppat.1010729.s011.pdf]

| S2 Table. List of plasmids used in this study | | | |
|---|---|---|---|
| **Name** | **Description** | **Resistance Marker** | **References** |
| pYS5 | Plasmid used for complementation in *B.anthracis*; AmpR in *E. coli*; KanR in *B. anthracis* | Kanamycin, Ampicillin | [1] |
| pYS5-*codY* $^{His6}$ | Plasmid expressing CodY with His6 tag under PA promoter in *B. anthracis* | Ampicillin, Kanamycin | This study |
| pYS5-*codY* $^{His6*}$ | Plasmid expressing CodY with His6 tag under PA promoter in *B. anthracis* (BAS Δ*prkC*) | Ampicillin, Spectinomycin | This study |
| pYS5-*codY* $^{S215A}$ | Plasmid expressing phosphoablative CodY under PA promoter in *B. anthracis* | Ampicillin, Kanamycin | This study |
| pYS5-*codY S215A*$^{His6}$ | Plasmid expressing phosphoablative CodY with His6 tag under PA promoter in *B. anthracis* | Ampicillin, Kanamycin | This study |
| pYS5-*prpN* | Plasmid expressing PrpN under its own promoter in *B. anthracis* | Ampicillin, Kanamycin | This study |
| pProExHTc | *E. coli* expression vector with N-terminal His6- tag | Ampicillin | Invitrogen |
| pPROEXHTc-*codY* | Expression of His6-CodY in *E. coli* | Ampicillin | This study |
| pPROEXHTc-*codYS215A* | Expression of His6-CodYS215A in *E. coli* | Ampicillin | This study |
| pPROEXHTc-*codYS215E* | Expression of His6-CodYS215E in *E. coli* | Ampicillin | This study |
| pPROEXHTc-*prpN* | Expression of His6-PrpN in *E. coli* | Ampicillin | This study |

| | | | |
|---|---|---|---|
| pPROEXHTc-*prpC* | Expression of His6-PrpC in *E. coli* | Ampicillin | This study |
| pPROEXHTc-*atxA* | Expression of His6-AtxA in *E. coli* | Ampicillin | This study |
| pGEX5X3-*prkCcat* | Expression of GST-PrkC catalytic domain in *E. coli* | Ampicillin | This study |
| pETDuet-*prkC+codY* | Dual expression of His6-CodY and PrkC catalytic domain in *E. coli* | Ampicillin | This study |
| pETDuet-*codY* | Expression of His6-CodY in *E. coli* | Ampicillin | This study |

## Reference


1. Singh Y, Chaudhary VK, Leppla SH. A deleted variant of Bacillus anthracis protective antigen is non-toxic and blocks anthrax toxin action in vivo. J Biol Chem. 1989;264(32):19103-7. Epub 1989/11/15. PubMed PMID: 2509473.
