# Supplementary material for: Role of serine/threonine protein phosphatase PrpN in the life cycle of Bacillus anthracis
Source: PLoS Pathog. 2022 Aug 1;18(8):e1010729. doi: 10.1371/journal.ppat.1010729 (PMC9371265; doi:10.1371/journal.ppat.1010729)
Supplement: S3 Table — (PDF) [file ppat.1010729.s012.pdf]

S3 Table. Bacterial strains used in this study			
**Name**	**Genotype**	**Resistance marker**	**References**
DH5α	*E. coli F⁻ endA1 glnV44 thi-1 recA1 relA1 gyrA96 deoR nupG purB20 φ80dlacZ ΔM15 Δ(lacZYA-argF)U169, hsdR17*($r_K^- m_K^+$), λ^-	-	Invitrogen
BL21(DE3)	*E. coli* B strain*: F⁻ ompT gal dcm lon hsdS*$_B$($r_B^- m_B^-$) *λ(DE3 [lacI lacUV5-T7p07 ind1 sam7 nin5]) [malB⁺]*$_{K-12}$(λ^S)	-	Invitrogen
SCS110	*E. coli* SCS110 is an *endA⁻* derivative of the *JM110* strain *rpsL (Strr) thr leu endA thi-1 lacY galK galT ara tonA tsx dam dcm supE44 Δ(lac-proAB) [F´ traD36 proAB lacIqZΔM15]*	-	Stratagene
B. anthracis Sterne 34F2 (BAS)	*B. anthracis* strain pXO1$^+$, pXO2$^-$	-	NIAID, NIH
BAS Δ*prpN*	Null mutant *prpN* strain in BAS background	-	This study
BAS Δ*prpN*::*prpN*	pYS5-*prpN* in BAS Δ*prpN* background	Kanamycin	This study
BAS WT::*codYHis*$_6$	pYS5-*codY*^{His6} in BAS WT background	Kanamycin	This study
BAS Δ*prpN*::*codYHis*$_6$	pYS5-*codY*^{His6} in BAS Δ*prpN* background	Kanamycin	This study
BAS WT:: *codYS215AHis*$_6$	pYS5-*codYS215A* ^{His6} in BAS WT background	Kanamycin	This study
BAS Δ*prpN::* *codYS215AHis*$_6$	pYS5-*codYS215A* ^{His6} in BAS Δ*prpN* background	Kanamycin	This study
BAS Δ*prpN*:: *codYS215A*	pYS5-*codYS215A* in BAS Δ*prpN* background	Kanamycin	This study
BAS Δ*prkC*::*codYHis*$_6$	pYS5-*codY*^{His6} in BAS Δ*prkC* background	Kanamycin, Spectinomycin	This study
