# Supplementary material for: Role of serine/threonine protein phosphatase PrpN in the life cycle of Bacillus anthracis
Source: PLoS Pathog. 2022 Aug 1;18(8):e1010729. doi: 10.1371/journal.ppat.1010729 (PMC9371265; doi:10.1371/journal.ppat.1010729)
Supplement: Attachment — Submitted filename: Response to reviewers.docx [file ppat.1010729.s013.docx]

**Role of serine/threonine protein phosphatase PrpN in the life cycle of *Bacillus anthracis***

Aakriti Gangwal^1^, Nitika Sangwan^1¶^, Neha Dhasmana^1¶^, Nishant Kumar^1,2^, Chetkar Chandra Keshavam^1^, Lalit K. Singh^2,#a^, Ankur Bothra^4^, Ajay K. Goel^3^, Andrei P. Pomerantsev^4^, Stephen H. Leppla^4*^ and Yogendra Singh^1*^

**RESPONSE TO REVIEWER’S COMMENTS**

The authors would like to thank the reviewers for their comments. We have taken care of all the points mentioned by the reviewers to improve the manuscript. We have performed most experiments suggested by the reviewers and have included the data in the manuscript main text or supplementary file. The revised manuscript text is highlighted as Blue Font throughout the file and a clean version with modifications is uploaded as the manuscript file. Below are the specific responses to each reviewer’s comments:

**Response to Reviewer**

**Reviewer #1- (Page 1-2)**

**Major Issues: None**

**Minor issues:**

1. A more succinct manuscript could be had if sentences on the following lines were deleted (information could be re-routed to figure legends, materials and methods, supplement, or not included at all): a. 119-126, b. 356-361, c. 375-394 (these lines basically reiterate the results)

Response- As suggested by the reviewer, we have made appropriate corrections.

2. The schematics in Figures 2A, 7A, and 8A are rather simplistic and intuitive—I would suggest deleting them or moving to the supplement if you feel they are necessary. The model in Figure 10 could instead become the visual abstract instead of its own figure.

Response- As suggested, we have either removed or moved these figures to supplementary data.

3. Fig. 2C, Could it be mentioned why recombinant PrpN migrates slower in the gel?

Response- We thank the reviewer for pointing out this observation. The difference in mobility pattern is because of the presence of a hexa-histidine tag along with amino acids encoded by the plasmid (28 amino acids) in the recombinant protein resulting in an increase of around 3 kDa in its molecular weight.

4. Fig. 3E, is there an understanding why a doublet band appears for PrpN in late stationary? And for Fig. 9B, is there an understanding as to why LF produces three bands in WT but only 1 predominant band in the codY S215A mutant?

Response- We thank the reviewer for pointing out this observation. Even we were intrigued by this pattern (Fig. 3E, now Fig 2E) and probably it could be due to some PTM of PrpN in late stationary growth phase. Further investigation is required to confirm this hypothesis. Multiple bands in LF in BAS WT could be processed forms of this protein in the system.

This could merely be an artifact, since these multiple bands were not observed when similarly, BAS WT was probed using anti-LF (Fig 5B and 5D).

**Reviewer #2- (Page 3-7)**

**Major Issues:**

| **Reviewer’s Comment** | **Author’s Response** |
| --- | --- |
| 1. What is the kinase involved in the phosphorylation of CodY? This point is never discussed in the text. Since the authors have recently published a manuscript on PrkC of B. anthracis and have a prkC mutant, the impact of PrkC inactivation on the level of phosphorylation of CodY in vivo should be tested. A phosphorylation test of CodY by PrkC in vitro could be also done. | To address this query, we performed an *in-vitro* kinase assay using catalytic domain of PrkC and CodY. Phosphorylation signal was checked by phospho specific Pro-Q diamond stain and anti-pSer antibody. Additionally, CodY produced in *E. coli* using pETDuet vector system containing PrkC was also checked by Pro-Q to examine CodY phosphorylation status. Overall, these results confirmed PrkC mediated phosphorylation of CodY protein. This information is provided in **Fig 6E, 6F** and **S5A**.  As suggested, to test this *in-vivo*, we probed CodY produced in *prkC* mutant strain and compared the phosphorylation level w.r.t. CodY produced in BAS WT using anti-pSer. Our results, however showed no difference in pCodY suggesting regulation of CodY phosphorylation by other STKs. This data is explained in the manuscript and illustrated in **S5B Fig**. |
| 2. 1. (First Part)  The authors showed that the level of phosphorylation of CodY increased in the prpN mutant in B. anthracis. However, they did not demonstrate that CodY is a direct PrpN target and that the site of phosphorylation of CodY in Bacillus anthracis is Ser215. They should complete their study by an analysis of the purified CodY they obtained from B. anthracis (WT and ?prpN strains) by Mass spectrometry to confirm that this serine is the phosphosite targeted by PrpN. Alternatively, a CodYSer215A-His6 could be expressed in B. anthracis and purified by the same strategy than the wild-type copy of CodY.  (Second Part)  To confirm that the effect of PrpN on CodY is direct, the addition of the purified PrpN to the CodY purified in vivo followed by a western-blot with an antipSer should be done. In addition, to estimate the purification of CodY, a gel should be added to western blot in Figure 7B. | Since, the background bacillus strains we used for CodY production were not protease deficient, some modifications were made in the purification protocol affecting the overall % purity of CodY protein in the eluted fractions (**Fig 6A**). Nevertheless, we tried mass spectrometry analysis of these proteins and phosphorylation site was detected at Ser215 residue but with low confidence.  Alternatively, as suggested by the reviewer to confirm ser215 phosphosite- CodYS215A produced in bacillus strains (BAS WT and BAS Δ*prpN*) was probed using anti-pSer. No signal was detected in these phosphoablative mutants, indicating phosphorylation of CodY specifically at ser215 (**Fig 7A**). Additionally, these phosphoablative mutants were resolved on a Phos-tag gel along with native CodY to confirm the above results. Only one band was observed in phosphoablative mutants, while native CodY showed multiple bands (**Fig 7B**).  A ser/thr phosphatase assay was performed using purified PrpN and CodY produced in bacillus strain (*in-vivo*). Instead of an immunoblot, this information is alternatively provided in **Fig 6D**.  Also, reproducing this experiment on immunoblot is tricky since the difference in the molecular mass of recombinant CodY(32kDa) and PrpN (30kDa) is marginal, resulting in poor separation on gel.  As suggested, purification gel images of CodY produced in BAS WT and BAS Δ*prpN* are added in the figure panel (**Fig 6A**). |
| 2.2. Finally, we would also suggest to use PhosTag gel combined with the Anti-CodY antibody. This PhosTag system, which leads to a separation of phosphorylated and unphosphorylated forms of proteins will allow to estimate more precisely the proportion of the CodY protein phosphorylated in your different strains. | We thank the reviewer for introducing us to this technique. As suggested proteins were resolved on a Phos-tag gel and probed using anti-CodY. This data is provided in **Fig 7B**. |
| 2.3. The impact of prpN inactivation on other phenotypes related to CodY should be tested (motility, biofilm formation, heme as iron source…). Since CodY controls an operon involved in autolysis and chain separation in Bacillus cereus (Huillet et al, Plos one 2016), we would suggest to analyze the morphology of the strains carrying a phosphoablative mutation of CodY you have obtained. | Since, *B. anthracis* unlike other Bacillus genus strains is a non-motile bacterium, we did not examine the strains for motility. Role of PrpN in biofilm was examined and the null mutant strain showed defective biofilm formation (**S8 Fig**). As suggested, we also examined the morphology of bacillus strains overexpressing CodYS215A. Overexpression of CodYS215A in BAS Δ*prpN* showed a considerable reduction in twisting and bending of bacterial chains (**S9 Fig**). We monitored these strains at stationary growth phase as well to examine the role of this mutant in chain separation. No difference in chaining phenotype was observed. Unexpectedly, overexpression of CodYS215A in BAS WT resulted in bacterial coiling (data not shown). One possible explanation for this phenotype could be the disturbance in CodY phosphorylation status. This hypothesis requires further investigation and we will be examining this in our future studies. |

**Other comments:**

Line 127-134. This PrpN phosphatase is present in the B. cereus group and in B. subtilis. It is not clear if this phosphatase is conserved in other Firmicutes. This point should be indicated.
Figure 2 should be a supplementary Figure.

Response- As suggested, evolutionary relationship of PrpN in firmicutes was examined by phylogenetic analysis (**S2 Fig**). The results of this analysis are explained in the accompanying text. Fig 2 is moved to supplementary figures. It is now mentioned as **S3 Fig**.

Line 189-191 and Figure 3E: In the western blot with the anti PrpN antibody, two bands seem to be present for the point IV (late stationary phase). This point should be indicated and discussed.

Reviewer #1 also asked about this. Please see-Page 2 above for response. We have added this information in the manuscript.

Line177: the prpN mutant entered the stationary phase earlier as indicated but had also a reduced growth yield compared to the WT strain. This point should be added.

Response- As suggested, we have added this information.

Line 216: the authors indicated that “a high population of multi-septa cells in the BAS ?prpN strain”. It would be better to determine the proportion of cells with multiple septa. It would be more precise than a high population.

Response- As suggested, multi-septated cells were quantified and the values are now represented in **Fig 3D**.

Figure 3D, 4A and 5A. The quality of the optical microscopy pictures is very poor. New figures of better quality should be provided. It is difficult to analyze the data as it is.

Response- We have changed/modified the mentioned microscopic images.

Role of PrpN in the sporulation process. The authors showed that prpN inactivation led to a drastic drop in spore formation. However, the sporulation does not seem to be blocked at a particular step. These results should be more carefully described and discussed.

Response- As suggested, we have modified our explanation.

Discussion
A recent study has shown that CodY plays a role in the control of sporulation in Bacillus anthracis (Gopalani et al, BBRC, 2016). Indeed, the overexpression of codY in B. anthracis led to a 100-fold repression of sporulation strongly suggesting that CodY is a repressor of sporulation. This reference should be cited. prpN inactivation led to a decreased sporulation efficiency and not to a sporulation derepression as expected from the increased CodY phosphorylation. This suggest that the effect of PrpN on sporulation depends on a regulator or factor downstream of CodY in the sporulation cascade. This point should be more carefully discussed. The possible relative role of PrpC and PrpN in the B. cereus group should be discussed as well as hypothesis concerning the factors likely involved in CodY phosphorylation.

Response- The information is now provided in the discussion section.

**Minor points:**
Line 175 : deleted “by measuring the absorbance of 600 nm until 14 hours”
Line 176: delete “to ensure axenic bacterial culture”
Line 206-207: Split the sentence. Indicate, Interestingly, these variations increased at stationary phase synchronizing with a higher PrpN production.
Line 254-260 corresponds to materials and methods. This part should be deleted. Some details could be indicated in the Figure legend if necessary.
I would suggest to replace protein expression by protein production in all the text.

Response- The suggested changes are made in the manuscript.

**Reviewer #3- Page 8-9**

| **Reviewer’s Comment** | **Author’s Response** |
| --- | --- |
| 1. The EMSA shift data showing mutants of CodY fails to bind DNA is convincing. However, there are a few issues with it first the binding affinities are nearly 100 times higher than other reported binding affinities. This could be in part due to not using GTP and Branched chain amino acids or use of SYBR green vs. P32 labeled probes. | As mentioned by the reviewer, the difference in binding affinities (without effectors) is due to the detection method used in our study. These experiments were initially performed using P32 labeled probes and similar binding affinities as reported earlier were observed, but later due to unavailability of radioactive facility in the host department, we switched to a lesser sensitive detection technique- fluorescence. |
| 2. Second they do not test binding in the presence of GTP or branch chained amino acids which enhance DNA binding of CodY. It may be important to know how DNA binding is affected in the presence of the signals that promote DNA binding. | We thank the reviewer for this valuable comment as it provided additional information regarding the role of ser215 in CodY binding affinity. We inluded more data points of CodY protein concentration for better analysis and as suggested performed EMSA in the presence of GTP and BCAA. This experiment was repeated in the absence of GTP and BCAA, for comparative analysis. As reported earlier, our results showed enhanced binding of CodY in the presence of effector molecules. Interestingly, CodYS215A showed higher binding affinity than native CodY suggesting the involvement of a charge based interaction between CodY and its target DNA. The data is showed in **Fig 7C** and **S7C**. |
| 3. Third it may be helpful to perform circular dichromism with the mutants of CodY S215 to determine if they are folded properly particularly S215E which shows no binding. | We agree with the reviewer, since CodYS215E showed complete abrogation in its DNA binding ability, misfolding of protein could be a possible reason. To test this, as suggested these proteins were subjected to CD analysis and showed properly folded proteins (**S7B Fig**). |
| 4. The authors have done such thorough work in this study it is surprising they did not test the effect of the CodY S215A mutation on toxin expression. | We did check the effect of CodYS215A mutation on toxin expression and it was provided previously in Fig 9. Overexpression of CodYS215A in the prpN null mutant strain partially reverted toxin expression, indicating CodYS215A as a positive regulator toxins synthesis in *B. anthracis*. This figure is **Fig 8** in the modified manuscript. |
| 5. Figure 9 needs statistics | As suggested the figure is updated. This figure is **Fig 8** in the modified manuscript. |

**Minor comments:**

1. Figure 3 it would be helpful to show the mutants at these different time points as well.
Response- As suggested, we have added microscopic images of mutant strain as well. This is shown in **Fig 2D**.

2. Please show complement for Figure 4.

Response- We have updated this figure. It is shown in Fig **3C**.
